# Extended Microbiological Characterization of Göttingen Minipigs in the Context of Xenotransplantation: Detection and Vertical Transmission of Hepatitis E Virus

**DOI:** 10.1371/journal.pone.0139893

**Published:** 2015-10-14

**Authors:** Vladimir A. Morozov, Alexey V. Morozov, Avi Rotem, Uriel Barkai, Stefan Bornstein, Joachim Denner

**Affiliations:** 1 Robert Koch Institute, Berlin, Germany; 2 Engelhardt Institute of Molecular Biology, RAS, Moscow, Russia; 3 Beta-O2 Technologies Ltd., Rosh Haain, Israel; 4 Center Internal Medicine, University Clinics Carl Gustav Carus, Technical University, Dresden, Germany; Duke University, UNITED STATES

## Abstract

Xenotransplantation has been proposed as a solution to the shortage of suitable human donors. Pigs are currently favoured as donor animals for xenotransplantation of cells, including islet cells, or organs. To reduce the xenotransplantation-associated risk of infection of the recipient the pig donor should be carefully characterised. Göttingen minipigs from Ellegaard are often used for biomedical research and are regularly tested by their vendor for the presence of numerous bacteria, fungi, viruses and parasites. However, screening for some pathogens transmittable to humans had not been performed.The presence of microorganisms was examined in Göttingen Minipigs by PCR methods. Since zoonotic transmission of porcine hepatitis E virus HEV to humans has been demonstrated, extended search for HEV was considered as a priority. RNA from sera, islet and other cells from 40 minipigs were examined for HEV using different real-time reverse transcription (RT)-PCRs, among them two newly established. In addition, sera were examined by Western blot analysis using two recombinant capsid proteins of HEV as antigens. HEV RNA was not detected in pigs older than one year including gilts, but it was detected in the sera of three of ten animals younger than 1 year. Furthermore, HEV was also detected in the sera of three sows six days after delivery and their offspring, indicating vertical transmission of the virus. PCR amplicons were cloned, sequenced and the viruses were found to belong to the HEV genotype (gt) 3/4. Anti-HEV immunoglobulins G were detected in one sow and maternal antibodies in her six day old piglet. Since Göttingen minipigs were negative for many xenotransplantation-relevant microorganisms, they can now be classified as safe. HEV may be eliminated from the Ellegaard herd by selection of negative animals and/or by treatment of the animals.

## Introduction

Xenotransplantation is an option to overcome the shortage of human transplants including islet cells. However, there are three principal hurdles on the way to clinical trials: first, the prevention of immune rejection, second, the achievement of physiological compatibility, and third, prevention of transmission of zoonotic microorganisms from the animal donor to the human recipient. In the past years numerous efforts were undertaken to generate multitransgenic animals to prevent rejection of the transplant, to evaluate microbiological safety of different pig breeds (for review see [1–3]) and to establish a “precautionary principle” strategy to combat unknown risks [4].

There are only a few specified pathogen free (SPF) porcine breeding facilities in the world. At present Auckland island pigs, produced in such a facility, are well characterized [5–7] and were used in approved clinical trials of pig islet cells transplantation to diabetic patients [8].

Göttingen minipigs are the result of crossbreeding the Minnesota minipig, the Vietnamese potbelly pig and the German Landrace pig. This breed is used in biomedical research and may be considered as donor of islet cells. The herd bred at Ellegaard, Denmark (http://minipigs.dk/the-goettingen-minipig/) is produced in a SPF facility. Physiologic parameters, health status and genetics of the pigs are well-defined [9, 10]. In addition, the animals are screened twice a year for 27 bacteria, 16 viruses, three fungi and four parasites (http//www.minipigs.dk/).

Most of the zoonotic microorganisms of the pig may be eliminated by SPF or designated pathogen-free (DPF) breeding of the animals. We have recently characterized a group of the Göttingen minipigs concerning the prevalence and expression of porcine endogenous retroviruses (PERVs) [11]. In fact, PERVs cannot be eliminated because they are integrated in the genome of pigs. PERV-A and PERV-B (can infect human cells) and PERV-C (infects only pig cells) [12–14] were found in Göttingen minipigs, but their expression was low [11].

One of the not yet fully analysed viruses in Göttingen Minipigs is the hepatitis E virus (HEV). HEV was first isolated from human cases of non-A and non-B hepatitis [15], later also from pigs, it is widely distributed [16]. HEV is the sole member of the genus *Hepevirus* of the family *Hepeviridae*. It is a small (30–35 nm in diameter) non-enveloped virus containing single-stranded, positive-sense RNA of 7.2 kb, which is capped and polyadenylated [17–19].

The genome contains three open reading frames (ORF 1–3). ORF 2 is coding for the capsid protein and this gene is frequently used as target for virus detection by PCR methods Depending on the expression system various sizes of the capsid protein can be detected [20–22]. However, the size of the capsid protein in the virus particle was calculated as 71 kDa, the glycosylated protein should not be greater than 79 kDa [23]. It has been shown, that amino acids 394–457 of the capsid protein form an immunodominant region (IDR) and an epitope binding neutralising antibodies has been identified as well [24, 25] The epitope binding the neutralizing antibodies is conformational and includes three regions (aa residues 496–499, 510–514, and 573–578) [26].

Four genotypes (gt) of HEV (gt1-4) have been described in mammals; these are further divided into a total of 24 subtypes [27]. HEV gt1 has five subtypes, gt2 has two subtypes, gt3 has ten subtypes, and gt4 has seven subtypes. Several new genotypes have recently been described in bats, rabbits, ferrets, rats, birds and fish [28, 29] and a new taxonomy was proposed, which subdivides the family *Hepeviridae* into two genus and five species (Orthohepevirus A-D and Piscihepevirus A) grouping HEV from humans, pigs, wild boar and deer, and some other mammals as Orthohepevirus A [28, 29].

HEV gt1 and gt2 are found only in humans and transmission occurs mostly through contaminated water, faecal-oral route, allotransplantation and blood donation [30–35]. Infection with HEV gt1 and gt2 is associated with sporadic hepatitis, but large outbreaks are rare [30, 36]. Hepatitis induced by these genotypes of HEV is typically a self-limiting disease without progression to chronic illness [30, 34]. However, HEV infection during pregnancy can be severe and result in fulminant hepatitis, still birth, premature delivery and an overall mortality rate of about 20–25% [30, 37–39].

The main reservoirs of HEV gt3 and gt4 are wild boars and domestic pigs [40–43]. The HEV gt3 is most frequent in the USA and Europe, while gt4 is found mainly in Asia [32]. Infected animals show no sign of disease [44, 45]. The immune response of wild boars and domestic pigs against HEV is variable and depends on the country, the area and the farm [33, 41, 42, 46, 47]. In most cases zoonotic transmission of HEV gt3 and gt4 to humans is the result of consumption of undercooked liver or meat from pork, wild boar or deer [48–51]. Infections of humans with HEV gt3 subtypes c, e and f are most common in Europe [27]. Infection of humans with HEVgt3 and gt4 is asymptomatic in the majority of cases; however, immunocompromised people are at risk of chronic hepatitis [30, 33, 34, 52, 53]. To summarise, HEVgt3 and gt4 are less virulent in humans than are HEV gt1 and gt2 [33, 34].

The presence of Jaagsiekte sheep retrovirus (JSRV), a beta-retrovirus, which is the causative agent of a contagious lung adenocarcinoma in sheep [54], was also investigated. JSRV is easily transmitted from sheep to goat and *vice versa*, but only one study examined the prevalence of JSRV in farm pigs [55].

Here we demonstrate the absence of 88 pathogens in three adult Göttingen minipigs and 15 pathogens in another three adult animals. It is well established that HEV in infected pigs remains latent and the viral load in animals may differ significantly [33, 41, 43, 56, 57]. As the genome of this virus is characterised by genetic variability, sensitive real-time RT-PCRs methods targeting different genomic regions, were used for virus detection. Testing of 40 animals of different ages showed that nine animals were infected with HEV, among them three mother sows and three of their six day old piglets, thus, indicating vertical transmission of HEV. Immunoglobulin G (IgG) antibodies against HEV were detected in one sow and maternal antibodies in her piglet. JSRV proviruses were not detected in the DNA from liver of minipigs when tested by nested-PCR. With this study a quite extended characterisation of the microbiological safety of the Göttingen minipigs was achieved.

## Materials and Methods

### Animal materials

Sera and tissues from Göttingen minipigs were obtained from Ellegaard Göttingen, Soroe Landevej 302, 4261 Dalmose, Denmark (http//www.minipigs.dk/). Ellegaard has permission from The Animal Experiments Inspectorate (often referred to as The National Authority in Denmark). The permission has the specific number: 2012-15-2934-00615. Originally the herd was founded by Caesarean section.

In the first setting sera from three minipigs (animals older than one year) were screened for 15 microorganisms at Zoologix (Chatsworth, CA USA, http://www.zoologix.com/) (S1 Table). In the second setting sera from three so called “retired breeders” (24–48 months old) were tested also at Zoologix for >88 microorganisms listed in Table 1.

**Table 1:** Microorganisms tested negative in three “retired breeders” minipigs, age 24–48 months.

Pathogens	Methods
Bacteria (20)	
Brucella abortus, B. microti, B. melitensis, B. pinnipedalis, B. suis, B. canis, B. ovis and B. neotomae	real-time PCR
Burkholderia mallei, pseudomallei	PCR
Chlamydophila felis / Chlamydophila psittaci	real-time PCR
E.coli	real-time PCR
Fusobacterium	real-time PCR
Bacillus anthracis	real-time PCR
Listeria monocytogenes	real-time PCR
M. tuberculosis, M. bovis, M. microti, M. intracellulare, M. avium, M. gastri, M. africanum, M. scrofulaceum, M. ulcerans, M. simiae, M. kansasii, M. chelonae, M. fortuitum, M. marinum, M. genavense, etc.	real-time PCR
*Actinobacillus pleuropneumoniae	real-time PCR
*Bordetella bronchiseptica	real-time PCR
*Brachyspira (Serpulina) pilosicoli	real-time PCR
*Campylobacter, Campylobacter jejuni, C. coli and C. lari	real-time PCR
*Leptospira	real-time PCR
*Pasteullera multocida	real-time PCR
*Salmonella	real-time PCR
*Yersinia enterocolitica	real-time PCR
*Clostridium perfringens	real-time PCR
*Erysipelothrix rhusiopathiae	real-time PCR
*Staphylococcus	real-time PCR
*Streptococcus	real-time PCR
Viruses (20)	
Nipah virus	reverse transcription / real time-PCR
Porcine cytomegalovirus	real-time PCR
Porcine lymphotopic herpesvirus 1 and 2, PLHV-1 and -2	real-time PCR
Rabies virus	reverse transcription / real-time PCR
Hepatitis E virus	reverse transcription / real-time PCR
PERV	reverse transcription / real-time PCR
*Encephalomyocarditis	reverse transcription / real-time PCR
*Rotavirus	reverse transcription / real-time PCR
Influenza virus, H5N1, H5N2, *H1N1, *H2N2, H3N8, H4N6, H7N7, H8N4, H9N2	reverse transcription / real-time PCR
BVDV	reverse transcription / real-time PCR
Swine fever virus	reverse transcription / real-time PCR
PHEV	reverse transcription / real-time PCR
Pseudorabies	real-time PCR
Porcine adenovirus	real-time PCR
Porcine circovirus type 1	real-time PCR
*Porcine circovirus type 2	real-time PCR
Porcine enterovirus	reverse transcription / real-time PCR
PRRSV	reverse transcription / real-time PCR
Swine pox virus	real-time PCR
Swine vesicular disease	reverse transcription / real-time PCR
Vesicular stomatitis virus	reverse transcription / real-time PCR
Nematode/worm (2)	
Taenia solium	real-time PCR
Trichinella spiralis	real-time PCR
Protozoa (3)	
Cryptosporidium	PCR
Trypanosoma cruzi	real-time PCR
*Toxoplasma gondii	real-time PCR
Fungi (4)	
Aspergillus	real-time PCR
Cryptococcus neoformans	real-time PCR
Microsporum	real-time PCR
*Candida albicans	real-time PCR
**Total: 49 groups of pathogens (>88 individual microorganisms)**	

Furthermore, EDTA whole blood and nasopharyngeal swabs, faecal swabs, and pancreas tissues were tested (see below). Fluorescent dye real-time RT-PCR and other PCR approaches were used for the detection of microorganisms. The sensitivity of detection RT-PCR methods varied between 10 and 100 copies. A total of 42 samples including 6 liver, 6 kidney, 28 sera and 2 islet cell specimens from 34 Ellegaard minipigs of different ages were tested at Robert Koch Institute (RKI) for the HEV infection. All samples arrived deep frozen and were stored at -80 C before processing.

### RNA and DNA extraction

RNA was isolated from liver and kidney using RNeasy mini kit (Qiagen, Hilden, Germany) and from 200 μl of sera using ZR viral RNA kit (Zymo Research Corp., Irvine, CA, USA), respectively. RNA was also isolated from pellets obtained by ultracentrifugation of 1.5 ml of sera diluted (1:3) in PBS (2.5 h at 32000 rpm, Ti50 rotor, Beckman, USA). The RNA samples were used in real-time RT-PCR immediately or were kept frozen at -80°C before processing. DNA from the liver was isolated using DNeasy Blood and Tissue kit (Qiagen GmbH, Hilden, Germany). The RNA and DNA were quantified on NanoDrop spectrophotometer.

### Primers and probes

**Fig 1:**
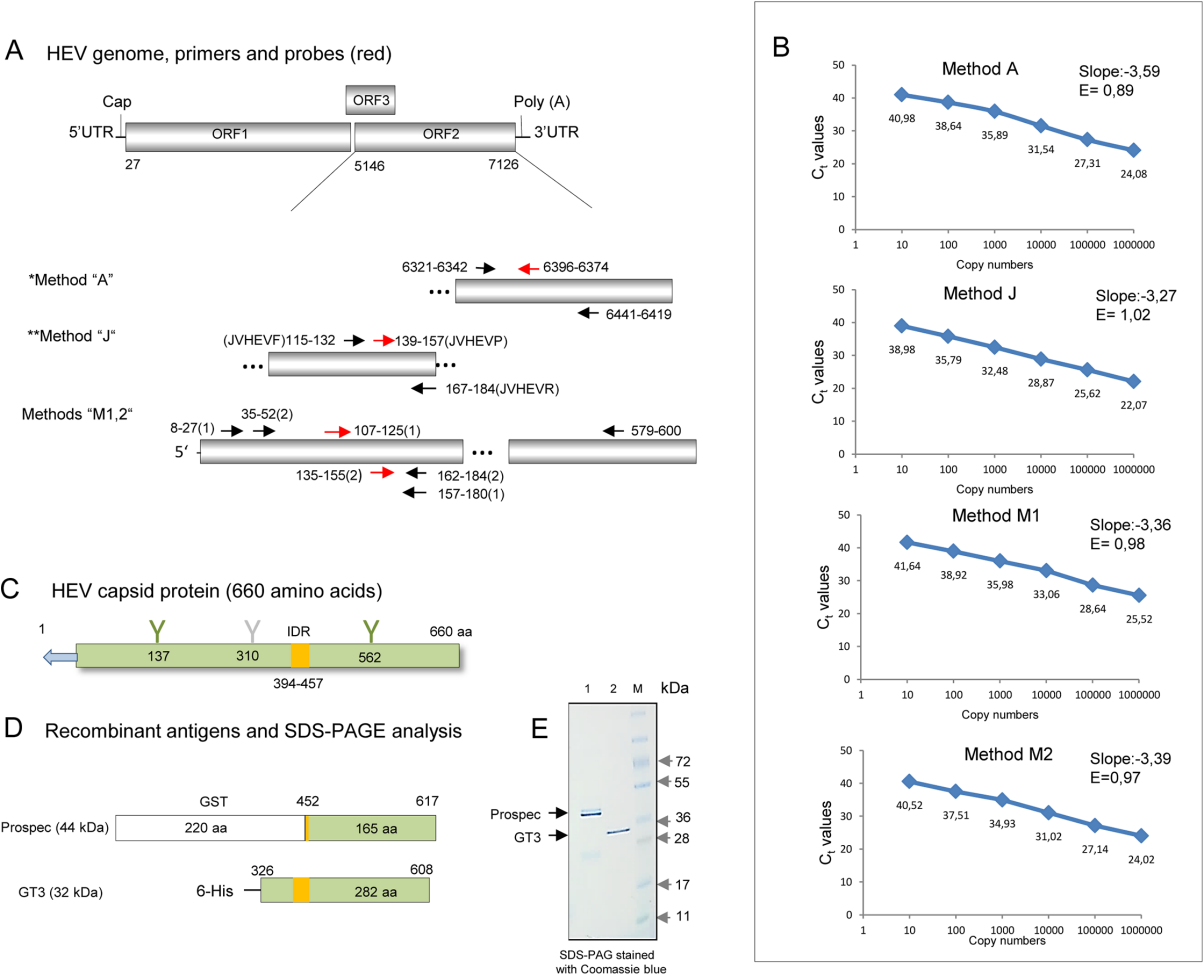
HEV genome, primers and probes, recombinant proteins and real-time RT-PCR parameters. (A) Schematic presentation of the HEV genome with three open reading frames (ORF 1–3). UTR—untranslated region. The numbers of nucleotides are given from the first nucleotide of ORF 2. Cap- cap structure, Poly (A)—poly A sequence. PCR primers (black arrows) and probes (red arrows) used in the real-time RT-PCR methods “A”, “J”, “M1”,and “M2” for the detection of HEV ORF2. Primers for methods M1 and M2 are given in brackets as 1 or 2, respectively. Numbers are given as in A. ^*^Method “A” was established by Adlhoch et al. [41]; **Method “J” was established by Jothikumar et al. [59]. (B) Parameters of the used real-time RT-PCR methods. Standard curves and PCR efficiencies are shown. One reference plasmid was used for the real-time RT-PCR method “A” and another, for the other three methods. (C) HEV ORF 2 is coding for the 660 aa long capsid glycoprotein. Putative glycosylation sites are marked in green, a low probability glycosylation site is marked in grey. Positions of the asparagine are numbered. The immunodominant region (IDR) is given in yellow. The signal peptide is shown as a blue arrow. (D) Recombinant proteins (Prospec and GT3) used as antigens in Western blot analysis are shown. GT3 contains the entire immunodominant region (IDR). The Prospec antigen contains glutathion-S-transferase (GST) on the N-terminus. GT3 contains a 6His-tag on the N-terminus. The fragments corresponding to the ORF2 are given in green. The numbers started from the first amino acids of the capsid protein. (E) Comparative analysis of the recombinant antigens by SDS-PAGE is shown on the right. 500 ng of proteins were loaded on the gel, the gel was stained with Coomassie brilliant blue. Lane 1 –Prospec, lane 2 –GT3, M–Size markers.

All primers and probes used in this study targeted ORF2 gene of the HEV (Fig 1A and 1B). Primers for the real-time (RT-PCR) and TaqMan PCR sequence-specific probes for methods “M1”, and “M2” were selected manually based on an alignment of 65 HEV gt1-4 sequences from the GenBank.

The primers and probes were analysed for the secondary structure and possible hairpins. Melting temperature was estimated by Sigma-Aldrich (Germany). All primers and probes for the real-time RT-PCR were synthesised by Sigma-Aldrich (Germany). A complete list of primers and probes is given in S2 Table.

### HEV standard and real-time RT-PCR methods

The WHO International Standard for HEV [58] was used to prepare the reference plasmid. In brief, the RNA was isolated, reverse transcribed using primers F8-27, R184-162 and the SensiFast probe no ROX one tube kit (Bioline, Luckenwalde, Germany). The 177 bp amplicon was cloned into pTOPO-TA (Invitrogen, Life Technologies, USA) and sequenced using BigDye Terminator sequencing kit (Life Technologies, USA). The plasmid was called “p177Ref.”. The copy number was estimated using the following formula: Number of copies = (amount * 6.022x10^23^) / (length * 1x10^9^ * 650).

The first real-time PCR method that has been used was established by Adlhoch et al. [41] and was called “A”. The method is based on generic primers and a specific probe that targeted the central part of ORF2 gene. The second method was established by Jothikumar et al. [59] and was called “J”. It targeted the ORF2–ORF3 overlapping region and was shown to be sensitive for the detection of different HEV genotypes (Fig 1A). The high efficacy and sensitivity of the system was confirmed by other laboratories [60, 61]. Third, two in-house reverse-transcriptase RT-PCRs methods were developed (“M1”, “M2”). “M1” was designed to amplify HEV gt3 and “M2” to amplify both the gt3 and the gt4. However, the efficacy of the detection of HEV gt4 by system “M2” was not tested. Both systems targeted the 5’-region of ORF 2, and allowed sequencing of the amplicons.

To establish a standard curve for the real-time PCR methods, two reference plasmids were used. The plasmids “p177Ref.” (see above) and pHEV RKI, containing a 121 nt fragment of ORF2 kindly provided by Marco Kaiser (Robert Koch Institute, Berlin, Germany) were used as references for the method “J”, “M1”, “M2” and “A”, respectively (Fig 1B). Duplex real-time RT-PCR assays for the detection of HEV and porcine cyclophilin A [62] transcripts were performed in 25 μl reaction mixture using SensiFast probe no ROX one tube kit as recommended by the supplier (Bioline, Luckenwalde, Germany). 500 ng or 250 ng of RNA extracted from liver and kidney (for 40 or 42 cycles of amplification, respectively) and 20 ng of total RNA from sera were used for amplification. Amplification programs for methods “A” and “J” were used as recommended by the authors [41, 59]. Amplification using methods “M1” and “M2” were performed under the following conditions: reverse transcription for 35 min at 48°C, denaturation at 95°C for 7 min and 40–42 cycles of amplification (kidney liver, islet cells) with denaturation at 95°C for 10 s, annealing at 59°C for 20 s and extension at 70°C for 30 s. 45 cycles of amplification were used for the detection of HEV in sera. Each real-time RT-PCR assay was accompanied by 10-fold dilution series of a standard plasmid. Reporter fluorescence was measured using an Mx3005P Multiplex Quantitative PCR System (Stratagene, La Jolla, CA, USA).

### Conventional semi-nested RT-PCR and nested PCR

Screening for HEV in liver and kidney of minipigs was also carried out using the Titan one tube RT-PCR system (Roche Diagnostics GmbH, Germany). Reverse transcription was performed at 45°C for 60 min with primers F(8–27) and R(600–579). After denaturation at 94°C for 2 min semi-nested PCR was performed with 3 μl from the reverse transcription cycle using primers F(35–52) and R(600–579) (S2 Table). The following program of 38 amplification cycles was used: denaturation at 94 ^0^C for 30 s, annealing at 56°C for 30 s and elongation at 68°C for 1 min. The last elongation was carried out for 7 min.

Primers for nested PCR targeting the gag and orf X regions of the JSRV genome are given in S2 Table. 500 ng of each DNA sample (equivalent to approximately 7,5x10^3^ cells) were tested using GoTaq green polymerase master mix (Promega, Madison, WI, USA). The parameters of the PCR were as follows: initial denaturation at 95°C for 2 min was followed by 38 cycles of amplification (95°C for 30 s, annealing for 53°C or 58 ^0^C (depending on primers) for 30 s and extension at 72°C for 45 s, final extension at 72°C for 10 min). For nested PCR 5 μl were taken from the first reaction and amplified using the same conditions as described above. To decrease possible cross-contamination by the positive control (goat genomic DNA) it was used only in the nested round.

### Cloning of the amplicons

After electrophoresis on 1.2% agarose gel the amplicons were extracted using Invisorb Spin DNA extraction kit (Stratec molecular, Berlin, Germany). The fragments were ligated into pCR 2.1-TOPO vector according to the protocol of the supplier (Invitrogen Life Technologies, USA). Z cells (Zymo Research, USA) were transformed with the constructs for 25 min on ice and plated on LB agar/ampicillin dishes for 18 hours at 37°C. Five clones from each dish were collected and amplified in LB/ampicillin medium overnight at 37°C. Plasmids were isolated using PureYield plasmid miniprep system (Promega, Madison, WI, USA) and cleaved with EcoR1 to check for the inserts. The clones were sequenced in both directions using primers from the cloning kit and BigDye terminator v.3.1 (Applied Biosystems, Germany). Cloned viral sequences from two minipigs were submitted to the GenBank (Submission ID 1790586).

### Antigens, PAGE and Western blot analyses

Two related antigens representing parts of capsid protein were used as targets in Western blot analyses (Fig 1C, 1D and 1E) First, a recombinant genotype 3 (GT3) ORF 2—HEV antigen (aa 326–608, GT3-Ctr, 32 kDa) [42] containing the immunodominant region and second, a recombinant 44.5 kDa protein with a glutathione-S transferase (GST) tag fused to the ORF2 fragment (aa 452–617) (Prospec, Ness Ziona, Israel). Electrophoresis of the antigens was performed in precast preparative Tris-Glycine 4%–20% gradient SDS-PAGE gels using SDS Tris-Glycine sample buffer (Novex, Life Technologies Carlsbad, CA, USA). Proteins were transferred for 2 h at 4°C (46V) onto supported nitrocellulose (Protran BA83, Whatman GmbH, Dassel, Germany). The membrane was stained with Ponceau red and cut into strips. The load of antigens per strip was estimated as 300 ng. The membranes were blocked with 6% dry milk in PBS with 0.1% Tween 20 (blocking buffer) overnight at 4°C. Strips were treated with sera diluted 1:150 in blocking buffer for 2 h at room temperature. Goat anti-pig IgG (Abcam, UK) or goat anti-human IgG alkaline phosphatase conjugated antibodies (Sigma-Aldrich, USA) were taken 1:1000 in blocking buffer. Reaction was developed using NBT (nitro-blue tetrazolium chloride)—BCIP (5-bromo-4-chloro-3'-indolyphosphate p-toluidine salt) substrate (Promega, Madison, WI, USA). Sera from a HEV infected pig and an HEV infected patient, and non-infected pigs were used as positive and negative controls, respectively.

### Software

The Blast program (NCBI) was used for database search. The sequence alignments and neighbour-joining phylogenetic analyses were performed using software package Lasergene Version 10 (DNASTAR, Inc. Madison, USA).

## Results

### Extended analysis of microorganisms in Göttingen minipigs

To analyse the prevalence of porcine microorganisms in Ellegaard Göttingen minipigs in more detail, three animals older than 1 year were screened in addition to the regularly analysed microorganisms (http//www.minipigs.dk/) for 15 additional pathogens by fluorescence based real-time PCRs (S1 Table). None of these microorganisms were found. In a second setting the list of pathogens was extended to include 49 additional groups of microorganisms (20 bacteria, 20 viruses, two nematodes/worms, three protozoa and four fungi), counting at least 88 single microorganisms (Table 1). In this setting three retired breeders (sows of the age of 24–48 months) were examined. The animals were screened using fluorescence based real-time PCR and real-time RT-PCR methods. HEV and CMV were included in both settings. All animals were found negative for all tested microorganisms. Some of the microorganisms were among those regularly tested by Ellegaard (http//www.minipigs.dk/).

### Real-time RT-PCR methods for detection of HEV

Four real-time RT-PCR methods were used for the HEV detection in minipigs in addition to the method used at Zoologix. Two of these have been described [41, 59]. The first method, here called “A”, targeted the central part of the HEV ORF 2 and the second one, here called “J”, targeted the ORF2-ORF3 overlap (Fig 1B, S2 Table). Since amplicons from the two PCR reactions were very short and not suitable for extended sequencing, two new RT-PCRs methods were established,”M1” and “M2”. The “M1” and “M2” RT-PCR reactions produced amplicons of 172 bp and 148 bp, respectively. Sensitivity of RT-PCR methods “J”, “M1”, “M2” was tested using serial ten-fold dilutions of the reference plasmid “p177Ref.” that was prepared from the WHO international HEV standard (gt3a). The threshold cycle (C_t_) values were plotted against genomic equivalent copies (Fig 1B). Linear regression slope values for the three systems were: for A = -3.59, for J = - 3.27, for M1 = - 3.36, for M2 = - 3.39. The efficiency (E) of the PCR was estimated using the following formula: E = -1+10^(-1/slope)^. The systems “A”, “M1” and “M2” demonstrated very close parameters of amplification (Fig 1B). However, method “J” demonstrated superior efficiency and required 2–3 cycles less to achieve the 10 copy limits. The quantification experiments were repeated twice.

However, when the real time RT-PCR using serial dilutions of the RNA from the WHO International HEV standard was performed, the detection limit of the real-time RT-PCR “J” method was shown to be 10 copies, while that of methods “A”, “M1” and “M2” was about 150–200 copies. This suggests that the difference in the detection limits between the four real-time PCR methods depends on the size of the amplicons which determines partially the efficacy of the reverse transcription step.

### Detection of HEV

Having in mind that the prevalence of HEV in pigs is age dependent [46, 57, 63, 64], we analysed pigs of different ages. In addition to the six animals tested at Zoologix (three in the age groups over one year and three between 24 and 48 months) additional 34 animals from the Göttingen minipig SPF facility of four different age groups (Groups 1–4). were examined Initial analyses were performed on twelve adult animals. Six sera and two islet cells specimens from six so-called “retired breeders” (Table 2) and tissues from six randomly selected adult one year old minipigs born from different parents (Table 3).

**Table 2:** Group 1. Adult minipigs “retired breeders”. Analysis of sera (s) and islets cells (i).

Animal	Gender	Age (years)	Samples	RT-PCR J (C_t_)	RT-PCR M2 (C_t_)	Western blot
308175	F	2	s, i	neg, neg	neg, neg	neg
305383	F	4	s, i	neg, neg	neg, neg	neg
216646	F	3	s	neg	neg	neg
219748	M	2	s	neg	neg	neg
318208	F	1,5	s	neg	neg	neg
220407	F	1,5	s	neg	neg	neg
**Total: 6**				0/6, 0/2	0/6, 0/2	0/6

**Table 3:** Group 2. Adult minipigs. Analysis of kidney (k) and liver (l).

Animal	Gender	Age (years)	Samples	RT-PCR A (C_t_)	RT-PCR J (Ct)	RT-PCR M 1, 2 (Ct)
217288	M	1	k, l	neg, neg	neg, neg	neg, neg
313266	F	1	k, l	neg, neg	neg, neg	neg, neg
217275	M	1	k, l	neg, neg	neg, neg	neg, neg
217271	M	1	k, l	neg, neg	neg, neg	neg, neg
217200	F	1	k, l	neg, neg	neg, neg	neg, neg
313578	F	1	k, l	neg, neg	neg, neg	neg, neg
**Total: 6**				0/6, 0/6	0/6, 0/6	0/6, 0/6

**Fig 2:**
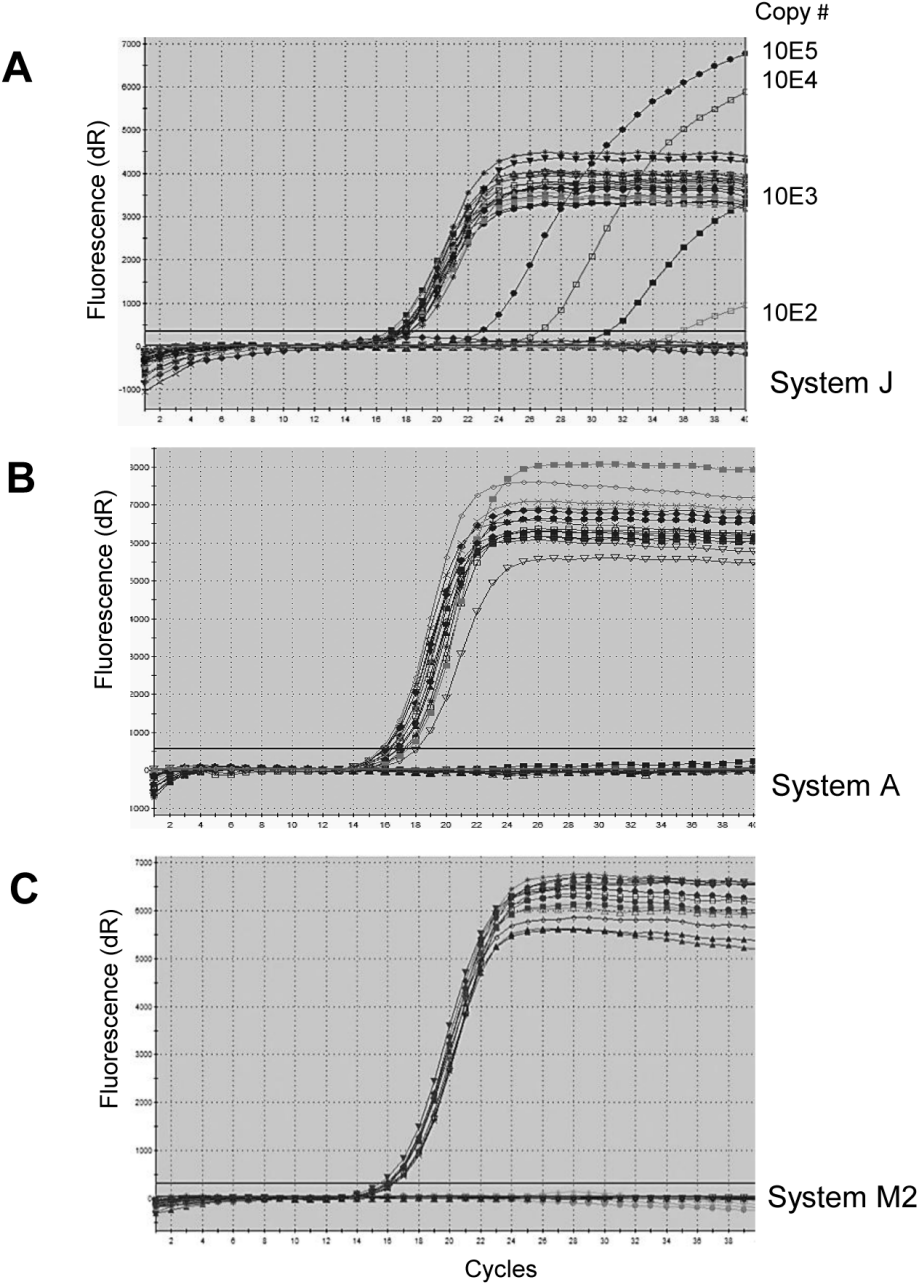
Lack of HEV in liver and kidney tissues from adult Göttingen minipigs. Three different duplex real-time RT-PCR systems “A”, “J”, and “M2” were used to screen for HEV in liver and tissue of the animals from Group 2 using 500 ng of total RNA for the analysis. Porcine cyclophilin A gene was used as a house keeping control and equal load of RNA was confirmed. Serial 10-fold dilutions (10E5-10E2) of the reference plasmid were used as a control for the system “J”. Analyses were performed in duplicates. In addition, minipigs from Group 2 were also found negative when RNA specimens were tested using a conventional semi-nested RT-PCR (data not shown).

It is worth mentioning, that islet cells from animals of Group 1 had been used for pig to non-human primate xenotransplantations. RNA specimens extracted from islet cells and sera from animals of Group 1 were tested using the monoplex real-time RT-PCR methods “J” and “M2”. RNA from kidney and liver from animals of Group 2 were tested by duplex real-time RT-PCRs methods “A”, “J”, and “M1, 2” with a porcine cyclophilin A as housekeeping gene (Fig 2).

Since, the adult animals from Group 1 and Group 2 (from different pens) were found negative in all real-time RT-PCR assays (Tables 2 and 3), it is possible to suggest that animals older 1 year in the Ellegaard SPF facility are not infected. Group 3 was composed of 9–10 months old fatteners (Table 4).

**Table 4:** Group 3. Fatteners (~ 10 months old). Analysis of sera (s).

Animal	Gender	Age (days)	Sample	RT-PCR J (C_t_)	RT-PCR M2 (C_t_)	Western blot
315007	M	311	s	(41.01)	neg	neg
315172	M	296	s	(39.88)	neg	neg
218268	M	308	s	neg	neg	neg
218277	M	308	s	neg	neg	neg
218278	M	308	s	neg	neg	neg
314995	F	313	s	neg	neg	neg
315032	F	309	s	neg	neg	neg
315048	F	308	s	neg	neg	neg
218337	F	301	s	neg	neg	neg
320203	F	325	s	(38.30)	n.t.	neg
**Total: 10**				3/10	0/9	0/10

n.t.- not tested.

RNA specimens from sera were tested by the monoplex real-time RT-PCR methods “J”, “M1”, and “M2”. Three out of ten animals were found positive by RT-PCR method “J”. The C_t_ values were 41.01, 39.88, and 38.30, respectively (Table 4). The C_t_ values allowed estimating the presence of 100–200 HEV RNA copies (or 5–10 RNA copy/ng HEV). Since 20 ng of RNA were used in a single reaction and 60 ng of RNA were isolated from 200 μl serum, the total amount of RNA containing particles (not obligatory infectious) in sera was about 1.5-3x10^3^/ml.

Given that the Göttingen minipigs were produced in a SPF facility with superior hygienic standards and taking into account that the 50% pig infectious dose (PID_50_) is high (about 1x10^4,5^/ml) [45], the chance of horizontal transmission of HEV would be extremely low. Therefore, vertical transmission of HEV remains the only option, as shown in humans with HEV gt1 [37, 38].

To investigate this assumption, six sow-offspring pairs were selected and were included in Group 4 (Table 5).

**Table 5:** Group 4. Sow-piglet (S-P) pairs. Analysis of sera (s).

Sow-piglet	Piglet gender	Age (years, days)	Sample	RT-PCR J (Ct)	RT-PCR M2 (Ct)	Western blot
**Subgroup 4.1**						
S305527-P319428	M	3y4m/48d	s	neg-neg	neg-neg	neg-pos*
S314674-P319340	M	1y4m/52d	s	neg-neg	neg-neg	neg-neg
S214145-P221670	F	2y5m/36d	s	neg-neg	neg-neg	neg-neg
**Subgroup 4.2**						
S314253-P320282	F	1y8m/6d	s	(33.90)-(40.62)	(31.72)-neg	pos-pos**
S314451-P320263	F	1y6m/6d	s	(36.82)-(38.76)	(38.83)-neg	neg-neg
S315784-P320257	F	1y2m/6d	s	(37.03)-(38.72)	(40.70)-neg	neg-neg
**Total: 12 (6+6)**				6/12	3/12	1(1*)/12

*- indeterminate result

**- Anti-HEV IgG immune response detected in S-P pair.

The animals in Group 4 were further subdivided based on the age of the piglets. Sows and their offspring older than 36 days were in Subgroup 4.1 and sows and their six day old offspring formed Subgroup 4.2. (Table 5). RNA specimens from sera were analysed in duplicates using real-time RT-PCR methods “J” and “M2”. Three sows and their offspring from Subgroup 4.1 were HEV-negative. In contrast, in Subgroup 4.2 all six animals were found positive by real-time RT-PCR “J” and three sows were also positive by real-time RT-PCR “M2”. As estimated by C_t_ values, the virus loads in sows were rather high from 5x10^3^ to 2x10^5^copies/ml. These values were at least four times higher than those of their offspring and also higher than those observed in two fattening minipigs from Group 3.

### Immunological screening for HEV

To analyse whether the Göttingen Minipigs produced antibodies against HEV, in-house Western blot analyses using two recombinant proteins corresponding to the HEV capsid protein were used.

**Fig 3:**
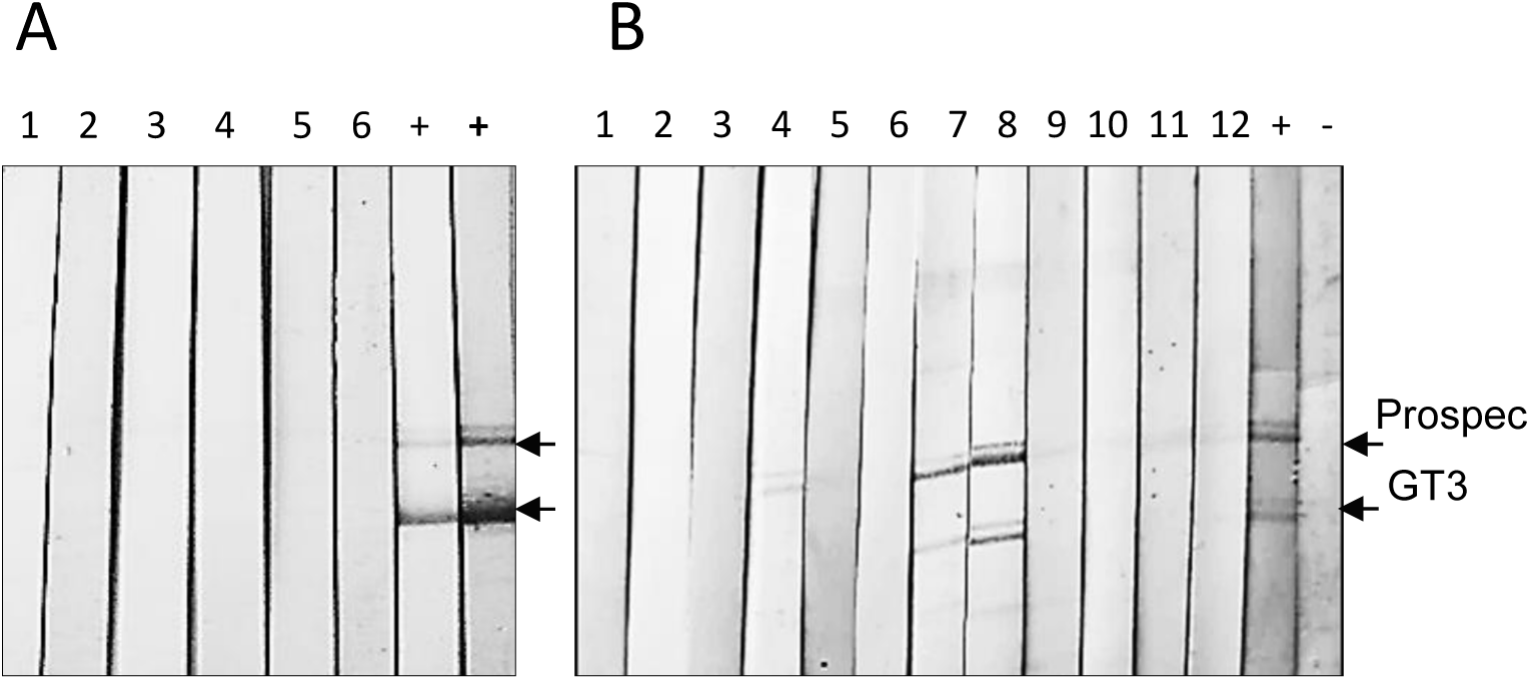
Western blot analysis of sera from Göttingen Minipigs. (A) Western blot analysis of sera from retired breeders (Group 1). The numbers on strips corresponded to the order of animals in Table 2. “+”–serum from a HEV infected pig diluted 1:300; “+ (bold)” serum from infected pig diluted 1:150. The antigen load was 300 ng/strip. Animal sera were tested twice in dilution 1:150. (B) Analysis of sera from sow-piglet pairs (Group 4). Odd numbers 1, 3, 5, 7, 9, 11 –strips incubated with sera from sows; even numbers 2, 4, 6, 8, 10, 12 –strips incubated with sera from piglets. Strip 4 was incubated with serum of piglet #319428. Strips 7, 8 (underlined) were treated with serum of the sow #314253 and serum of piglet #320282, respectively. “+” -serum from HEV infected pig; “-” serum from non-infected pig. The antigen load was 300 ng/strip. The sera specimens were diluted 1:150.

The first was a 32 kDa recombinant protein corresponding to the central part (aa 326–608, including the entire immunodominant region) of the capsid protein of HEV gt3 (GT3-Ctr) with a 6His-tag at the N-terminus [42]. The second, a 44.5 kDa recombinant protein (Prospec) contained the 452–617 aa fragment of the capsid protein tagged with GST. The antigen from Prospec contained only 6 aa of the immunodominant region. SDS-PAGE analyses of the antigen from Prospec showed a double band (Fig 1E) and these proteins were related as was confirmed by Western blot analysis using an anti-HEV reference serum (Fig 3A and 3B).

Both antigens were used simultaneously in order to decrease the probability of false-positive results. Sera reacting with both recombinant HEV antigens were considered as positive and sera reacting only with one antigen were treated as indeterminate.

Two groups (Group 1- retired breeders and Group 3—fattening pigs) were found negative for anti-HEV IgG. Analysis of sera from retired breeders is shown (Fig 3A). Sera from fatteners including sera from RT-PCR-positive animals #315007, #315172 and #320203 were also negative (Data not shown). Tests were repeated twice. Sera from adult minipigs of Group 2 were not accessible. An antibody response against two antigens was found in one adult animal from Group 4, Subgroup 4.2. Sera from sow #314253 and her six day old offspring (#320282) (Fig 3B), showed an IgG response against both core antigens. Evidently, the immune response observed in piglet #320282 represented maternal immunity. It is worth mentioning, that sow #314253 had not only a strong anti-HEV IgG response, but also showed the highest virus load as measured by real-time RT-PCR using two independent RNA extraction methods. The other HEV-positive sow-offspring pairs were seronegative. A weak reaction, but only with the antigen from Prospec (indeterminate reactivity), was detected in serum from a 48 days old piglet (#319428). In fact, the sow and this piglet were real-time RT-PCR negative.

Several sera reacted weakly with other proteins in the Western blot analysis, probably detecting bacterial contaminations of the recombinant core proteins.

### Analyses of the ORF2 HEV sequences from Göttingen minipigs

**Fig 4:**
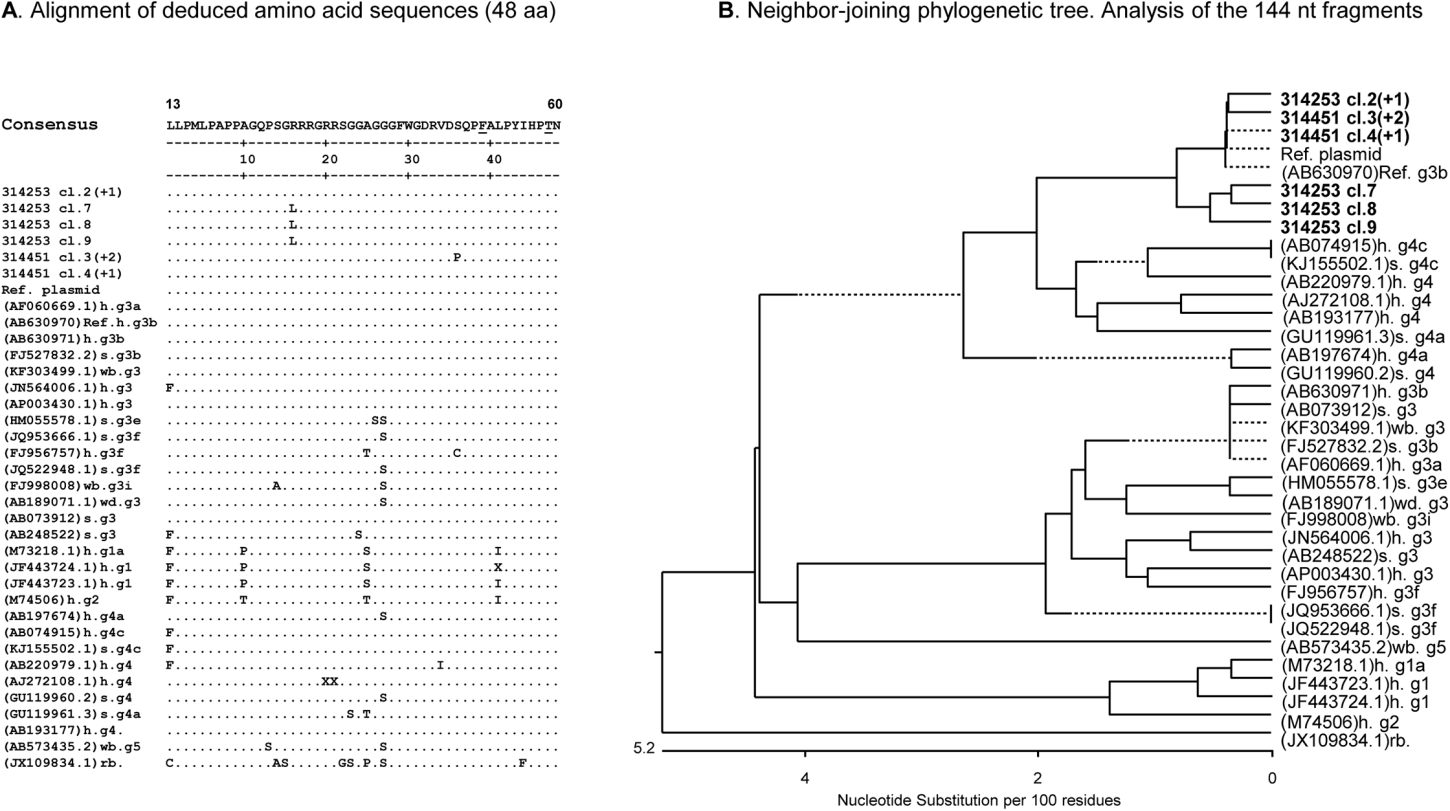
Analysis of the HEV sequences revealed in two sows. (A) Neighbour-joining phylogenetic tree of nucleotide sequences corresponding to a 144 nucleotides region in ORF2. Sequences from the infected sows #31453 and #314451 are given in bold. After the animal number the number of identical clones derived from the same amplicon is given in brackets as (+n). Accession numbers from the GenBank sequences are indicated. The origin of the sequences is indicated as: h-human; s-swine; wb-wild boar; rb-rabbit. The genotype and subgenotype are given as a number and a small letter, respectively. Absence of the letter means that the subgenotype is unknown. Brackets mark the HEV genotypes 3 and 4 (indicated as gt3 and gt4), respectively. The reference (Ref.) plasmid was designed using WHO standard of HEV (Acc. #AB630970). Nucleotide substitutions per 100 nucleotides are indicated. (B) Alignment of deduced amino acid sequences (48 residues) of cloned HEV sequences from sow #314451 and sow #314253 using Clustal W software. Amino acids substitutions associated with reduced infectivity of HEV (underlined in the consensus sequence) were not detected in the cloned sequences.

For analysis of the virus sequences, amplicons obtained by real-time RT-PCR “M2” using the RNA from sows #314253 and #314451 were re-amplified by conventional PCR using the same set of primers and cloned. Five clones from each amplicon were sequenced in both directions. After sequence editing the 144 bp long fragments were aligned with a set of HEV ORF 2 sequences of different genotypes (Fig 4).

Analysis of pair distance between clones 7, 8, 9 from sow #314253 and three identical clones, which were designated clone 3(+2), from sow #314451 demonstrated 97.9% identity with the reference plasmid. Comparison of clones 7, 8, 9 with clone 3(+2) showed 95.8% identity. The neighbour-joining phylogenetic analysis showed that nucleotide sequences from cloned amplicons clustered in one group with HEV gt4. It should be mentioned that the 5’-region of ORF 2 is not well suited for discrimination between gt3 and gt4. Thus, HEV gt3b (Acc. AB630970) from a Japanese blood donor was also present is this group (Fig 4A). Furthermore phylogenetic analysis demonstrated that HEV sequences derived from the Göttingen minipigs were related, but were not identical. In addition, a minor diversity between different clones from one amplicon was shown. In the absence of re-infection the presence of quasi-species favours Suggest a long time presence of the virus. Next, alignment of deduced amino acid (aa) sequences (aa position 13–60 of the capsid protein) also demonstrated some diversity between the clones (Fig 4B). For instance, three of five clones from sow #314253 contained a R28L substitution and three of five clones from sow #314451 contained a S48P substitution. Both mutations had not been observed previously in HEV. It remains to be determined how these substitutions influence particle morphogenesis and virus infectivity. The other 2 clones from each amplicon were identical to a set of gt3a and gt3b isolates, and also to a gt4 isolate (Acc. AB193177) from a Japanese patient with acute hepatitis. Interestingly, F51C and T59A substitutions which are usually associated with virus attenuation were not found in the capsid protein [64] (Fig 4B, underlined). Since the analysed sequences were different from the sequence of the reference plasmid prepared from the International WHO standard (Acc. AB630970), contamination with the reference plasmid can be excluded.

### Screening for JSRV proviruses

To detect JSRV in Göttingen minipigs, a nested PCR using primers targeting the *orfX* regions of the virus and DNA isolated from the liver was performed. No JSRV proviruses were found (S1 Fig), indicating that the animals were neither infected, nor contained endogenous sequences.

A similar result was obtained when primers targeting *gag* were used (data not shown). This confirms previous negative results in pigs performing Southern blot hybridization and Western blot analyses [55].

## Discussion

To ensure the microbiological safety of pig cells, including islet cells, tissues or organs to be used for xenotransplantation, a careful analysis of the donor must be performed.

Extended screening of Göttingen Minipigs from the Ellegaard SPF facility for previously untested pathogens (Table 1) failed to detect 88 different microorganisms in adult pigs—potential donors for islet cells for xenotransplantation. Since HEV detection is a difficult task and there are limited and conflicting data regarding HEV infection in minipigs from research facilities [60, 65]. We performed extensive examination of HEV infection in animals of different ages and sexes. It was surprising to find HEV in a SPF biomedical facility (Table 6.). In contrast, previously, 15 minipigs from research facilities in Japan were found HEV negative including absence of IgG or IgM immune responses against the capsid proteins of HEV gt1, gt3 and gt4 [65]. However, another study demonstrated that 100% (7 of 7) of 56 days old Göttingen minipigs were HEV infected [60]. Unfortunately, this investigation was not accompanied by serological testing and data on the relationships between the animals (e.g., the same litter?) and on housing of the animals was not provided.

**Table 6:** Summary table. HEV in minipigs.

Minipigs/samples tested	Age (months, days)	Group	RT-PCR positive/tested	Western blot positive/tested
**Tested at RKI**				
Retired breeders/islets, sera	18–48 months	Group 1	0/6	0/6
Sows soon after delivery/sera	16–40 months	Group 4. Subgroup 4.1	0/3	1*/3
Sows soon after delivery/sera	14–20 months	Group 4. Subgroup 4.2	3/3	1/3
Adult/liver, kidney	12–13 months	Group 2	0/6	n.a.
Fatteners/sera	9–10 months	Group 3	3/10	0/10
Piglets/sera	36–52 days	Group 4. Subgroup 4.1	0/3	0/3
Piglets/sera	6 days	Group 4. Subgroup 4.2	3/3	1**/3
**Tested at Zoologics**				
Retired breeders/blood	24–36 months		0/3	n.d.
Retired breeders/blood, pancreas, nasopharyngeal and faecal swabs.	24–48 months		0/3	n.d.
**Total minipigs tested: 40**			9/40	1+1** (1*)/28

*- indeterminate

**—maternal anti-HEV IgG; n.a.-not available; n.d.-not done.

Having sensitive detection methods it seems relatively easy to identify infected animals and to eliminate HEV from the pig herd. However, it is obviously more complicated for the following reasons: First, HEV infected pigs are asymptomatic [45], thus all animals in a herd should be tested. Second, HEVs of the same gt are characterized by genetic diversity. Third, during the long persistence in the herd, HEV might mutate. For example, analyzing HEV gt4 transmission among humans with hepatitis, a mutation rate of 1.4–1.7x10^-3^ nucleotide substitutions per site per year was estimated [49]. In *in vitro* experiments performing long-term consecutive virus passaging of HEV gt3 on human cells a higher mutation rate of 1.3–3.4x10^-3^ substitution per site per year was estimated [50]. Fourth, intergenotype and intragenotype recombination occurs including the 5’-region of the ORF2 in particular [66], re-infection and variability of the virus genome might hamper virus detection using generic primers and probes. For instance, the most efficient and broadly reactive real-time RT-PCR method, “J”, demonstrated about two log differences in efficacy when detecting different genotypes [59].

Samples from 40 Göttingen minipigs from the Ellegaard SPF facility were examined by real-time RT-PCR and by Western blot for HEV markers. The following age groups were included in the research: so called “retired breeders” (1,5–4 years old), adult animals (older 1 year), fattening pigs (about 10 months old), piglets (6–36 days old) and their mothers (older 14 months). Using four real-time RT-PCR methods HEV was not detected in tissues and sera of retired breeders and adult animals (older 1 year). However, three out of ten animals younger than 1 year and six out of 12 animals in a group of six sow-offspring pairs were infected with HEV (Group 4).

Sows six days after delivery were HEV-positive as shown by two real-time RT-PCR methods. Their offspring were positive too, but with a lower virus load. The difference in the virus load between sow and piglet varied between delta C_t_2 and delta C_t_6 (approximately from 4 to 64 times). Obtained positive results were confirmed three months later using sera from the HEV positive sows # 314253 and #314451. Interestingly, after that period the virus load in the sera of the animals was reduced by 4 and 8 times, respectively.

The 5’- region of the Orf2 gene is rather conservative, but it is not the region of choice for genotyping. Based on the alignment of the 144 bp sequence the genotype of the viruses was not clearly identified and it might be either gt3 or gt4. It is interesting to note, that the Vietnamese potbelly pig was one of the founder pigs of the Göttingen minipigs, but the prevalence of HEV gt4 in founder pigs was not investigated so far. Evidently longer sequences from more variable regions are required to make precise genotyping.

When the anti-viral immune response of HEV infected wild boars was analysed by ELISA, it was found either absent, low or high with no correlation to the overall virus load [42]. A significant diversity of immune response was also shown in infected domestic pigs [43, 44] and in pigs inoculated with capped RNA transcripts [64]. When analyzing pigs from 267 different herds in Germany, in 20% of the herds not a single animal was antibody positive, whereas in 23% of the herds more than 75% of the pigs were positive. A total of 1065 of 2273 sera (46.9%) were found to be anti-HEV IgG-positive. While 38.4% (306/796) of fatteners exhibited HEV-specific antibodies, 51.4% (759/1477) of sows exhibited anti-HEV antibodies [46].

Since standardized and approved serological tests are not available [28], an in-house Western blot was established using two overlapping recombinant HEV capsid proteins, and one of them contained the entire immunodominant region. It appears that the IgG immune response against these antigens was not frequent in minipigs from Ellegaard SPF facility Anti-HEV IgG were detected in the serum from sow S314253 (pair S314253-P320282) with the highest viral load and the serum from her six day old piglet, suggesting passive immunization. (Table 6). In this particular case the contribution of the anti-HEV maternal antibodies to the protection of the piglet was not investigated. In fact, the sow # 314253 was the only anti-HEV IgG-positive adult animal among five sows and six retired breeders tested.

A strong impact of maternal antibodies on preventing virus transmission was reported [67]. This antibody-mediated protection lasted between weeks 9 and 14 and frequently the decrease of maternal antibodies after week 6 was followed by increase of virus load [56, 68]. However, the antibody-independent decrease of virus load in farm animals after 5–6 months is difficult to explain [43, 63]. Nevertheless, HEV clearance by conformational neutralizing antibodies undetectable by peptide ELISA or Western blot analysis could not be excluded. To explain the lack of the immune response in certain infected animals, we suggest that infection of the fetus during pregnancy may result in development of immune tolerance against HEV. This immune tolerance might not only be restricted to minipigs in SPF facilities, but it might represent a common phenomenon among the farm pigs and the wild boars.

Follow-up studies performed on domestic pigs demonstrated that virus shedding was not detectable after day 120; however virus was still detectable at day 200 in internal organs of three of 13 pigs [63]. It remains to be determined whether the virus could be re-activated after 200 days or whether the virus is completely cleared in adult pigs and in adult Gottingen minipigs in particular.

Infection of pregnant women with HEV gt1 can result in severe disease [17, 30]. We suggest that in this case the disease progression and HEV gt1 replication may be triggered by pregnancy associated immunosuppression and/or overexpression of hormones (progesterone?). Involvement of progesterone in the activation of endogenous JSRV proviruses was demonstrated [69]. It is likely that in HEV infected pigs the mechanism of replication enhancement might be similar, but without clinical outcomes for the fetus and the sow. Recently, using negative strand-specific RT-PCR approach it has been shown that the placenta is a site of HEV gt1 replication in humans [70]. The data favor the probability of HEV gt3, gt4 replication in pig placentas and vertical virus transmission from sow to offspring.

Altogether, the Ellegaard minipigs are well characterised and may serve in future as donor pigs for xenotransplantation. 89 analysed microorganisms (including JSRV) were not found in adult Göttingen minipigs However, extended analysis of minipigs of different ages revealed HEV infections were in 3 animals younger than 1 year and in 3 sows-piglets pairs soon after delivery. It is likely, that—as in farm animals—the infection may be cleared or remains below the detection limit. It is possible that in in case of frequent pregnancies, infection remains detectable. Therefore, HEV-free donor pigs may be selected for xenotransplantation, possibly by treating them with Ribavirin [71] and/or vaccinating them with a HEV specific vaccine [72].

## Conclusion

Xenotransplantation using pig islet cells, tissues or organs may help to overcome the shortage of human transplants. Göttingen Minipigs are good candidates to be used for islet cell transplantation to treat diabetes. In order to achieve full microbiological safety, animals were screened for the presence of 89 bacteria, viruses, fungi and other microorganisms. They were found to be free of all tested microorganisms with exception of HEV. HEV was detected in young animals, in some sows soon after delivery and their offspring (Table 6) An anti-HEV humoral immune response was also reported in one mother and her offspring. These data demonstrate a mother to piglet transmission of HEV explaining the presence of the virus in a SPF facility with high hygienic standards. Therefore vertical transmission of HEV should be considered not only in SPF facilities producing pigs for medical purposes, but also in farms producing pork.

## Supporting Information

S1 Fig Lack of JSRV sequences in Göttingen minipigs. A nested PCR was performed using DNA from liver tissues and primers specific for JSRV orfX region. M–markers (GeneRuler 1 kb DNA ladder), lanes 1–6 –DNA from the liver of adult minipigs, lane 7 –DNA from a Large White pig, lane 8 –DNA from 293T cells, lane 9 –DNA from a goat (positive control). The positive control was used only in the second round of PCR amplification. The position of the amplicon is marked by an arrow.
(TIF)

S1 Table Additional microorganisms that were not detected in three 1 year old Göttingen minipigs. (DOC)

S2 Table Primers and probes. (DOCX)
