# Supplementary figures and images for: Extended Microbiological Characterization of Göttingen Minipigs in the Context of Xenotransplantation: Detection and Vertical Transmission of Hepatitis E Virus
Source: PLoS One. 2015 Oct 14;10(10):e0139893. doi: 10.1371/journal.pone.0139893 (PMC4605773; doi:10.1371/journal.pone.0139893)

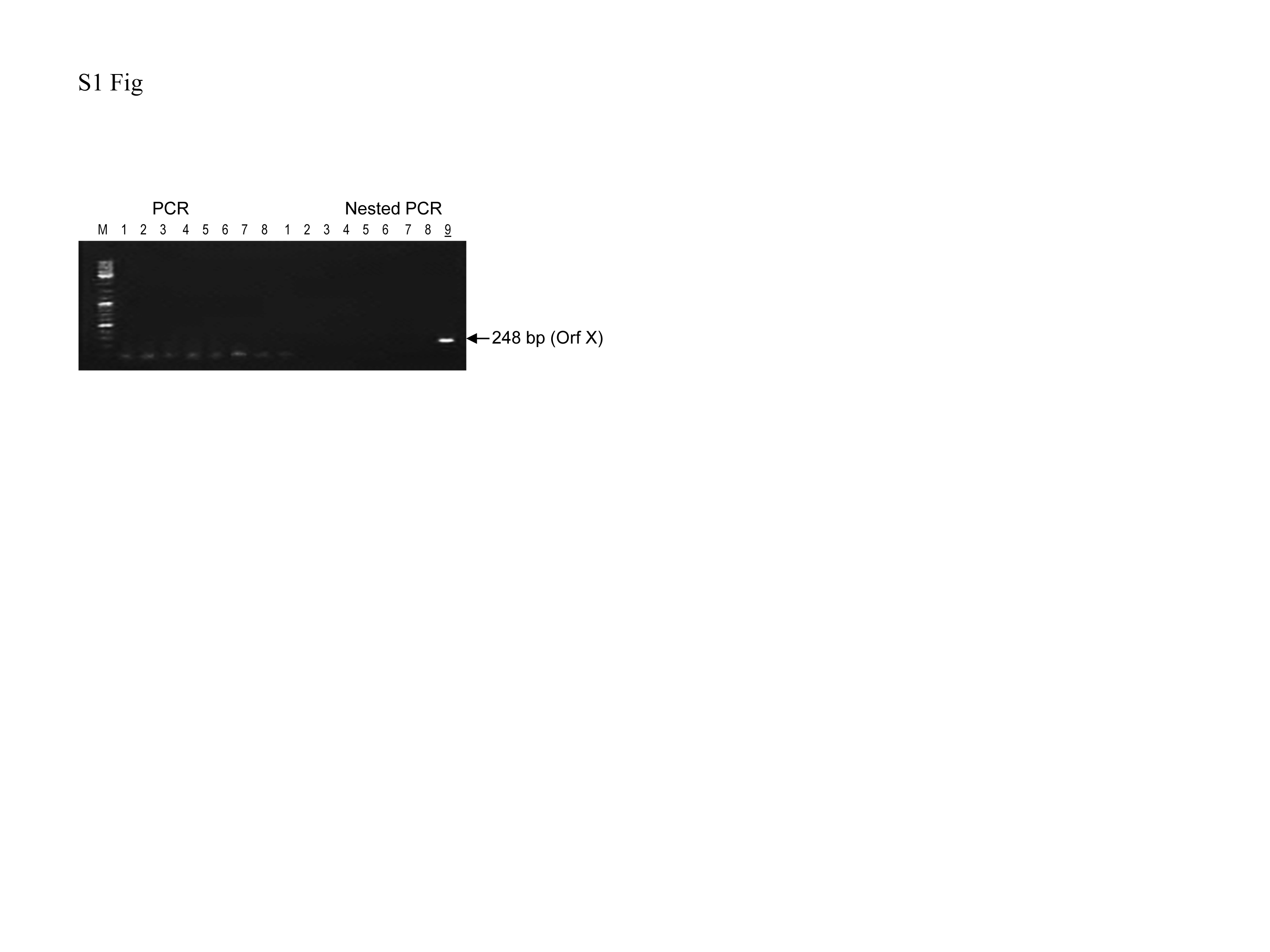

Supplement: S1 Fig — A nested PCR was performed using DNA from liver tissues and primers specific for JSRV orfX region. M–markers (GeneRuler 1 kb DNA ladder), lanes 1–6 –DNA from the liver of adult minipigs, lane 7 –DNA from a Large White pig, lane 8 –DNA from 293T cells, lane 9 –DNA from a goat (positive control). The positive control was used only in the second round of PCR amplification. The position of the amplicon is marked by an arrow. (TIF) [file pone.0139893.s001.tif]
