# Supplementary material for: Extended Microbiological Characterization of Göttingen Minipigs in the Context of Xenotransplantation: Detection and Vertical Transmission of Hepatitis E Virus
Source: PLoS One. 2015 Oct 14;10(10):e0139893. doi: 10.1371/journal.pone.0139893 (PMC4605773; doi:10.1371/journal.pone.0139893)
Supplement: S1 Table — (DOC) [file pone.0139893.s002.doc]

**S1 Table** Additional microorganisms that were not detected

in three 1 year old Göttingen minipigs.

| **Pthogens** | **Species** |
| --- | --- |
| **Bacteria (7)** | Brucella suis |
|  | Burkholderia (Pseudomonas) pseudomallei |
|  | Chlamydia psittaci |
|  | Escherichia coli O 157:H7 |
|  | Fusobacterium necrophorum |
|  | Listeria monocytogenes |
|  | Mycobacterium spp. |
| **Viruses (4)** | Porcine cytomegalovirus |
|  | Porcine lymphotropic herpesvirus |
|  | Rabies virus |
|  | Swine hepatits E virus |
| **Parasites (2)** | Cryptosporidium spp. |
|  | Trypanosoma cruzi |
| **Fungi (2)** | Aspergillus app. |
|  | Cryptococcous neoformans |
| **Total: 15** |  |
