# Supplementary material for: Extended Microbiological Characterization of Göttingen Minipigs in the Context of Xenotransplantation: Detection and Vertical Transmission of Hepatitis E Virus
Source: PLoS One. 2015 Oct 14;10(10):e0139893. doi: 10.1371/journal.pone.0139893 (PMC4605773; doi:10.1371/journal.pone.0139893)
Supplement: S2 Table — (DOCX) [file pone.0139893.s003.docx]

**S2 Table.** Primers and probes

| **Primers/Probes** | **Sequence 5‘-3‘** | **Nucleotides** | **Polarity** | **Detection methods** | **References** |
| --- | --- | --- | --- | --- | --- |
| F | 5’-CGACAGAATTGATTTCGTCGGC | 6321-6342 | + | A | * Adlhoch et al.[41] |
| R | 5’-CCYTTRTCYTGCTGNGCRTTCTC | 6441-6419 | - | A | * |
| JVHEVF | 5‘-GGTGGTTTCTGGGGTGAC | 5261-5278 | + | J | ** Jothikumar et al.[57] |
| JVHEVR | 5‘-AGGGTTGGTTGGATGAA | 5330-5313 | - | J | ** |
| F(8-27) | 5'-CTAGGGCTGTTCTGTTGCTG | 8-27 | + | M1 | M1 |
| R(180-157) | 5‘-GAGGGCGAAGGGCTYGAATC | 180-154 | - | M1 | M1 |
| F(35-52) | 5‘-TGCTTYTGCCTATGCTGC | 35-52 | + | M2 | M2 |
| R(184-162) | 5‘-AGGGGTTGGTTGGATGAATATAG | 184-162 | - | M2 | M2 |
| R(600-579) | 5‘-ATTTGGCACCAACGGGCGGTAA | 600-579 | - | nested |  |
| F cyclophilin | 5‘-TGCTTTCACAGAATAATTCCAGG | 174-196 | + | Cyclophilin | *** Duvigneau et al.[73] |
| R cyclophilin | 5‘-GACTTGCCACCAGTGCCATTA | 250-230 | - | Cyclophilin | *** |
| ORFX (4601) F1 | 5’-ATTGATGCAGCCCGAAAATC | 4601-4621 | + | JSRV |  |
| ORFX (4686) F2 | 5’-GCACGGCAAATTGTTAAATC | 4686-4705 | + | JSRV |  |
| ORFX (5091) R1 | 5’-TAATTCGTTGATGGGCTCGT | 5091-5042 | - | JSRV |  |
| ORFX (4889) R2 | 5’-TTCTCCAGTGTGAAGGGAAG | 4889-4870 | - | JSRV |  |
| gag F1 | 5’-GAAGAAGGTGGGAGAGCAAAT | 427-447 | + | JSRV |  |
| gag F2 | 5’-AAAAGGTCCCTGTCGAGACATT | 477-497 | + | JSRV |  |
| gag R2 | 5’-AGCTTGACAAGCAGAGTTAGCGT | 1600-1578 | - | JSRV |  |
| gag R2 | 5’-CCTATAGTATCTAAAAGTCGTGCCA | 1494-1519 | - | JSRV |  |
| Probe HEV TM | 6FAM-TYGGCTCGCCATTGGCYGAGAC-BHQ | 6396-6374 | - | A | * |
| Probe JVHEV | 6-FAM-TGATTCTCAGCCCTTCGC-BHQ | 5284-5301 | + | J | ** |
| Probe (125-107) | 6-FAM -CAGAAACCACCGCCGGCAC -BHQ | 125-107 | - | M1 |  |
| Probe (155-135) | 6-FAM-GCGAAGGGCTGAGAATCAACC-BHQ | 155-135 | - | M2 |  |
| Probe cyclophilin | Cy5-TGCCAGGGTGGTGACTTCACACGCC-BHQ | 205-228 | + | Cyclophilin A | *** |

*Method “A“(GenBank acc. #AB248520); **Method “J” (GenBank acc. # M73218); Methods “M” (GenBank acc. #KF303499); ***Cyclophilin A (GenBank acc. #FN401368); JSRV (GenBank acc. #NC001494).
